# Digital Interventions to Promote Self-Management in Adults With Hypertension: Protocol for Systematic Review and Meta-Analysis

**DOI:** 10.2196/resprot.4648

**Published:** 2015-11-20

**Authors:** Gary McLean, Elizabeth Murray, Rebecca Band, Kathryn Saunderson, Peter Hanlon, Paul Little, Richard J McManus, Lucy Yardley, Frances S Mair

**Affiliations:** ^1^ Institute of Health and Wellbeing College of Medical, Veterinary and Life Sciences University of Glasgow Glasgow United Kingdom; ^2^ E-Health Unit Research Department of Primary Care and Population Health University College London, London London United Kingdom; ^3^ Academic Unit of Psychology Faculty of Social and Human Sciences University of Southampton Southampton United Kingdom; ^4^ Primary Care and Population Sciences Faculty of Medicine University of Southampton Southampton United Kingdom; ^5^ Department of Primary Care Health Sciences University of Oxford Oxford United Kingdom

**Keywords:** digital intervention, hypertension, self-management

## Abstract

**Background:**

Digital interventions, defined as any intervention accessed and taking input from patients in the form of a computer/Web-based program or mobile phoned-based app, can potentially help empower patients to self-manage long-term conditions such as hypertension. Importantly, digital interventions have the potential to provide patients with personalized information and support for active involvement in treatment as well as cost saving.

**Objective:**

The purpose of this systematic review is to synthesize the evidence for using digital interventions to support patient self-management of hypertension, and determine their impact on control and reduction of blood pressure, other clinical outcomes, quality of life, medication adherence, health service utilization, and economic benefits.

**Methods:**

A systematic search of bibliographic databases including Medline, Embase, CINAHL, and PsycINFO will be undertaken. Abstracts and citations will be independently screened by 2 researchers against predetermined inclusion criteria. Any disagreements will be resolved by discussion and further consideration of the inclusion criteria. Only randomized controlled trials which have been published in peer peer-reviewed journals with a diagnosis of hypertension will be considered. Inclusion criteria will be (1) adults (age ≥ 18 years) with hypertension (as defined by the primary authors); (2) an interactive digital intervention compared with usual care; and (3) outcomes of objectively measured change in blood pressure. Data extraction from identified articles will be undertaken by 2 independent reviewers using a uniform template. The main outcomes are systolic blood pressure (SBP) and diastolic blood pressure (DBP), and quality of life indicators. Secondary outcomes include cost- effectiveness, medication adherence, emotional well-being, and physical activity. Risk of bias of included studies will be assessed using the Cochrane tool.

**Results:**

Our research is currently ongoing. Data will be summarized narratively, and if possible, meta-analyses will be performed to assess the impact of the interventions on outcomes.

**Conclusions:**

By summarizing and synthesizing available data, this review will help inform policy on the use of digital interventions for self-management of hypertension and will clarify areas for further research.

**Trial Registration:**

Prospero 2014: CRD42014010268; http://www.crd.york.ac.uk/PROSPERO/display_record.asp? ID=CRD42014010268 (Archived by WebCite at http://www.webcitation.org/6c5alQQJL)

## Introduction

Hypertension has been shown to be the highest attributable risk to death from cardiovascular disease, which is the leading cause of premature mortality worldwide [1]. Reducing levels of blood pressure, even by a small amount, can have a substantial effect on levels of mortality, particularly at the population level [2,3]. However, the rate of control and treatment of hypertension is suboptimal with a large gap found between detection and control [4]. Barriers to adequate blood pressure control include suboptimal treatment by clinicians, suboptimal monitoring due to availability barriers for both patients and clinicians, and suboptimal adherence to medication [5].

The success of blood pressure management depends, to a large degree, on the willingness and ability of the patient to change and maintain certain behaviors and adhere to medication regimens [6]. In England, the National Health Service (NHS) identified self-management as a major priority [7]. Self-management can encompass a wide range of behaviors in addition to medication use and monitoring of symptoms, such as an individual’s ability to manage physical, psychosocial, and lifestyle behaviors related to his/her chronic illness and appropriate use of medical care [8]. There is increasing interest in promoting the role of self-management, by which individuals take greater control over their own health and well-being, in supporting the management of long-term conditions such as hypertension [9]. Self-management in hypertension including self-titration and behavioral interventions has been shown to be effective [10-12]. In addition, self-management for hypertension can involve focusing on improving adherence to dietary approaches [13], weight loss [14], increased physical activity [15], smoking cessation [16], and moderation of alcohol intake [17]. A study exploring patients' experiences of an interactive mobile phone-based system designed to support the self-management of hypertension found that it helped them gain an understanding of the interplay between blood pressure and daily life, which resulted in increased motivation to follow treatment [18]. However, few family physicians, by whom most hypertension care is undertaken, have the infrastructure to support such interventions.

One potential method for improving self-management is through the use of interactive digital interventions, which offer the possibility of empowering patients to self-manage their long-term conditions, and by providing patients with better access to personalized information and support for active involvement in treatment, as well as producing significant savings in treatment costs [18-20]. The “interactive” aspect requires contributions from users to produce tailored material and feedback that is personally relevant. Interactive digital interventions are computer-based programs that can combine health information with behavior change, emotional and/or decision support to potentially improve the efficiency of health care by automating routine aspects of patient education, monitoring, and support, while improving services by giving patients convenient 24-hour access to detailed personalized feedback, and allowing health professionals to monitor patient status remotely [21,22]. It has been suggested that well-designed interactive digital interventions can be instrumental in changing patient health-related behavior, improve patient knowledge and confidence for self-management of health, which in turn can result in better health outcomes [11,12]. However, problems with the development and implementation of interactive digital interactions include cost and complexity [23] and high attrition rates (where patients do not use or make suboptimal use of the intervention) [24], respectively. If interactive digital interactions are shown to be an effective adjunct to treatment, further work will be required to address these challenges [25].

Examining the effect of interactive digital interventions in comparison to usual care is important as there is evidence that successful implementation depends on clearly demonstrating their benefits and cost effectiveness to clinicians [26,27]. Self-management interactive digital interventions in a primary care setting offer the opportunity of maximizing both reach and cost savings as the majority of those with hypertension are seen in a primary care setting. Although there are a number of reviews that have examined the impact of self-management in adults with hypertension [28-30], to our knowledge there are none that focus on self-management interactive digital interventions. Moreover, an overview of the literature [31] found 2 Cochrane reviews which concluded that while current evidence offered little support that self-monitoring and mobile phone messaging interventions provided benefit in supporting long-term illnesses, there is a need for further research into these issues [26,32]. Therefore, this systematic review aims to synthesize the evidence for using interactive digital interventions to support patient self-management of hypertension, and determine their impact on control and reduction of blood pressure, other clinical outcomes, quality of life, medication adherence, health service utilization, and health care costs.

## Methods

### Intervention and Self-Management

The term “digital intervention” can relate to a number of different types of intervention. For the purpose of this review it will include any intervention accessed through a computer (work or home), mobile phone, or other handheld devices, and include a Web-based program, desktop computer program, or apps that provide self-management information. Intervention participants may input information online or offline through the particular device used. The intervention must function without any directive input from health professionals, and be “interactive” in nature. We define “interactive” as requiring contributions from program users (eg, entering personal data and making choices) that alter pathways within the program to produce tailored material and feedback [33]. Studies that only involved sending blood pressure (BP) readings to a remotely located health professional and receiving advice about medication titration directly from a health professional will be excluded from this review. Interventions that included face-to-face contact and focused on medication adherence will be included if there is also an automated, interactive component without direct health professional mediation (ie, users report SBP interactively then receive automated messages advising them to increase/decrease medication as relevant to their BP levels; trial registration number CRD42014010268).

For the purposes of the review, we define a self-management support intervention as the care taken by individuals toward their own health and well-being comprised by the actions they take (1) to lead a healthy lifestyle, (2) to meet their social, emotional, and psychological needs, (3) to care for their long-term condition, and (4) to prevent further illness or accidents [34].

### Eligibility Criteria

Inclusion criteria, based on participants, interventions, comparisons, outcomes, and study design (PICOS acronym) [35] include (1) adult population (aged ≥ 18 years) with hypertension (as defined by the primary authors), (2) an interactive digital intervention (as defined earlier), (3) a comparator of usual care, (4) objectively measured changes in blood pressure (systolic or diastolic), (5) only randomized controlled trials (RCTs) as they present the strongest level of evidence, and (6) only studies published in journals and in English as evidence suggests that limiting studies in this way does not introduce significant bias [36].

### Search Methods for Identification of Studies

Searches will be undertaken by a professional systematic review company (York Health Economic Consortium). The search strategy is shown in Multimedia Appendix 1. The databases to be searched are Medline, Embase, CINAHL, PsycINFO, ERIC, Cochrane Library (including CDSR, DARE, Central, and HTA databases), DoPHER and TROPHI (both produced by the EPPI Centre), Social Science Citation Index, and Science Citation Index. These databases will be searched using a combination of subject headings, where available (such as MeSH), and words in the title and abstracts. The resources searched were chosen because they represent a reasonably wide range of core databases covering health care literature and were likely to contain the health care research that is relevant to the review eligibility criteria (RCTs published in peer-reviewed journals excluding literature and conference abstracts). We achieved coverage of journal articles about digital technology through searching the Social Science Citation Index and Science Citation Index.

The search strategy will combine the following concepts and study-type filter: (1) hypertension, (2) digital intervention, (3) self-management, and (4) RCTs.

Search terms for the intervention concept were informed by those used in a previous systematic review conducted on digital asthma self-management interventions [37]. To assess the robustness of the search strategy, PubMed was searched for relevant studies and we identified 10 relevant papers for potential inclusion. We then undertook a hand search of the journals from which the 10 studies were published (Circulation, Journal of American Medical Association [JAMA], American Heart Journal, Journal of Hypertension, Journal of Medical Internet Research, and Journal of Human Hypertension) but no further studies were found. The search strategy was then run to ensure it included the 10 studies among the 5606 papers it identified. The search will also be complemented by contacting experts in the topic under review and by carrying out citation searches for articles citing individual studies that are included in the review [38].

### Study Selection

Relevant studies will be ascertained by screening using Distiller software [39] with all identified studies assessed by 2 reviewers. Initially, abstracts will be screened and any potentially relevant studies will be identified and the full-text will be reviewed. Any inter-researcher disagreements over inclusion will be resolved by discussion and a possible third party if a consensus cannot be sought. Excluded studies will be listed with reason(s) for exclusion. The primary outcomes are changes in mean SBP and DBP and quality of life indicators (Table 1).

**Table 1:** Types of primary outcome measures.

Outcome measure description	Primary outcome	Secondary outcome
Clinical	Mean systolic and diastolic blood pressure	
	Quality of life indicators	
Cognitive		Self-efficacy
Behavioural		Medication adherence
		Dietary change
		Physical activity
		Alcohol intake
Affective		Depression
		Anxiety
		Emotional well-being
		Satisfaction with care
Economic		Health service utilization
		Costs of intervention

### Data Extraction and Management

Studies that meet the inclusion criteria will be screened in full by 2 reviewers working independently to extract relevant population, intervention, and outcome data using the Distiller software [39]. Inter-reviewer disagreements will be resolved by seeking consensus or decision by a third party. When papers with duplicate data are found, the largest dataset will be included in any meta-analysis.

### Assessment of Quality

Risk of bias will be assessed in each of the included studies by the 2 researchers working independently using the Cochrane collaboration tool for assessing bias [40]. The areas of bias that will be assessed include methods of allocation concealment, generation and presentation of allocation sequence, whether incomplete outcome data were assessed, and whether there was evidence of selective outcome reporting.

### Analysis

Details of the populations studied and each intervention will be presented in a table format describing patient and intervention characteristics. We will conduct a narrative synthesis describing, where possible, the components of the interventions including theoretical underpinning, what the mode of delivery was (eg, mobile phone, tablet, personal computer, or Web-based facilitation), how the information was uploaded (online/offline) and where (home/work/other), how ongoing engagement was encouraged, and how often it was used.

Where possible and appropriate we will undertake a meta-analysis that will compare changes between intervention and control participants in outcomes for which adequate data from a minimum of 3 studies are available. We will pool the data for each outcome using mean differences for continuous outcomes and relative risks for dichotomous outcomes. Studies of self-monitoring in hypertension have shown significant heterogeneity and so it is likely that that a random effects model will be required. This decision will be made following estimation of heterogeneity using the *I* ^2^ statistic (low <30%; moderate 30-75%; high ≥75%) [41]. Publication bias will be assessed, whenever possible (sufficient number of studies, low heterogeneity), using the Egger regression asymmetry test, the Begg adjusted rank correlation test, and visual examination of funnel plots [42,43]. If high levels of heterogeneity are shown to exist, we will conduct sensitivity analyses if the number of included studies allows, in order to investigate possible sources of heterogeneity including study quality (adequate versus inadequate allocation concealment, low versus high attrition) and sociodemographic factors that could act as effect modifiers (age, gender, and socioeconomic status).

Any subgroup analyses undertaken will be defined a priori. If the data permit, we will undertake the following subgroup analyses: (1) interventions that included self-monitoring of blood pressure versus those that did not, (2) mode of delivery (mobile phone versus other), and (3) primary goal of the intervention (reduction of blood pressure versus any other).

## Results

Our research is currently ongoing. Data will be summarized narratively and, if possible, meta-analyses will be performed to assess the impact of intervention on outcomes. The aim is to have all the results completed, written, and published by the beginning of 2016.

## Discussion

This review and proposed meta-analysis are part of a study aiming to investigate the best way of providing people with an interactive digital intervention for hypertension that can help them self-manage their health condition, with support as needed from health care professionals. It is thus important to assess previous research on digital interventions to support patient self-management of hypertension and assess the effects, if any, on control and reduction of blood pressure, other clinical outcomes, quality of life, medication adherence, health service utilization, and health care costs. The results of this review will aid our understanding of current knowledge in relation to the utility of digital self-management interventions for hypertension and identify important research gaps.

## Appendix

Multimedia Appendix 1 Search strategy for Ovid Medline(R) in-process and other non-indexed citations and Ovid Medline(R): 1946 to present.

## Abbreviations

BP: blood pressure
DBP: diastolic blood pressure
RCT: randomized controlled trial
SBP: systolic blood pressure
