# Supplementary material for: Digital Interventions to Promote Self-Management in Adults With Hypertension: Protocol for Systematic Review and Meta-Analysis
Source: JMIR Res Protoc. 2015 Nov 20;4(4):e133. doi: 10.2196/resprot.4648 (PMC4704904; doi:10.2196/resprot.4648)
Supplement: Multimedia Appendix 1 [file resprot_v4i4e133_app1.pdf]

1 exp Hypertension/ (107147)
2 hypertension$.ti,ab,kf. (124026)
3 exp Anti-Hypertensiontic Agents/ (249481)
4 or/1-3 (363394)
5 (computer or computers).hw. (454944)
6 exp computers/ (69513)
7 exp Computer Systems/ (138826)
8 Online Systems/ (7170)
9 Medical Informatics/ (8298)
10 Medical Informatics Applications/ (2043)
11 Decision Support Techniques/ (12588)
12 Educational Technology/ (1110)
13 Electronics, Medical/ (6191)
14 Audiovisual Aids/ (6223)
15 Telecommunications/ (4315)
16 Multimedia/ (1503)
17 Hypermedia/ (393)
18 Video Games/ (2255)
19 Electronic Health Records/ (6675)
20 exp Cellular Phone/ (4953)
21 Social Networking/ (816)
22 exp Telemedicine/ (15920)
23 Telenursing/ (116)
24 Telephone/ (9298)
25 Information Systems/ (18115)
26 Ambulatory Care Information Systems/ (1161)
27 Software/ (79787)
28 Mobile Applications/ (136)
29 Wireless Technology/ (1022)
30 Electronic Mail/ (1886)
31 (computer$ or microcomputer$ or pc or pcs or mac or macs).ti,ab,kf. (301621)
32 (phone$1 or mobile$1 or smartphone$ or handset$ or hand-set$ or handheld$ or hand-held$).ti,ab,kf. (76517)
33 ((electronic$ or digital$ or device$) adj2 tablet$).ti,ab,kf. (136)
34 ((digital$ or electronic$ or communicat$) adj2 device$).ti,ab,kf. (4965)
35 device-based.ti,ab,kf. (1421)

36 (device$ adj2 technolog$).ti,ab,kf. (1120)
37 (PDA or PDAs or personal digital).ti,ab,kf. (6770)
38 mp3-player$.ti,ab,kf. (89)
39 (online or on-line or internet or www or web or website$ or webpage$ or local area network$ or broadband or broad-band).ti,ab,kf. (146394)
40 (wireless or wire-less or wifi or wi-fi or GPS or global positioning system$ or bluetooth$).ti,ab,kf. (22232)
41 (text messag$ or texting or texter$1 or texted or SMS or short messag$ or multimedia messag$ or multi-media messag$ or mms or instant messag$).ti,ab,kf. (7826)
42 (social media$ or facebook or twitter or tweet or tweets).ti,ab,kf. (2188)
43 (webcast$ or webinar$ or podcast$ or wiki or wikis or youtube or you tube or vimeo).ti,ab,kf. (1372)
44 (app or apps).ti,ab,kf. (13618)
45 ((electronic$ or digital$ or device$) adj2 application$).ti,ab,kf. (2910)
46 (iphone$ or i-phone$ or ipad$ or i-pad$ or ipod$ or i-pod$ or palm os or palm pre classic$).ti,ab,kf. (1062)
47 (android$ or ios or s40 or symbian$ or windows).ti,ab,kf. (14115)
48 (samsung or nokia or apple$ or zte or lg or huawei or tcl communication$ or lenovo or sony or motorola or audiovox or utstarcom or siemens or blackberr$ or casio or cect or coolpad or fujitsu or htc or just5 or kyocera or lumigon or micromax or mitsubishi or modu or nec or neonode or openmoko or panasonic or pantech or philips or qualcomm or sagem or sanyo or sierra or sk teletech or soutec or trium or toshiba or vidalco).ti,ab,kf. (30522)
49 (video$ or dvd or dvds).ti,ab,kf. (77503)
50 (email$ or e-mail$ or electronic mail$).ti,ab,kf. (10632)
51 (chat room$1 or chatroom$1).ti,ab,kf. (254)
52 (blog$1 or blogging or blogger$ or weblog$1).ti,ab,kf. (762)
53 (bulletin board$1 or bulletinboard$1 or messageboard$1 or message board$1).ti,ab,kf. (405)
54 (software$ or soft-ware$).ti,ab,kf. (88011)
55 (interactiv$ or inter-activ$).ti,ab,kf. (34342)
56 (ehealth$ or e-health$ or mhealth$ or m-health$ or m-learning).ti,ab,kf. (2363)
57 (electronic learn$ or e-learn$).ti,ab,kf. (1276)
58 tele$.ti,ab,kf. (110507)

59 ((digital$ or electronic$ or communicat$ or information$) adj2 technolog$).ti,ab,kf. (12590)
60 ((digital$ or electronic$) adj (intervention$ or therap$ or treatment$ or medicine or medical$ or health$)).ti,ab,kf. (13013)
61 (ICT or ICTs).ti,ab,kf. (2895)
62 medical informatics.ti,ab,kf. (1891)
63 (remot$ adj3 (care or caring or cared or manag$ or consult$ or monitor$ or measur$)).ti,ab,kf. (3040)
64 or/5-63 (1252272)
65 (action plan or action plans).ti,ab,kf. (3381)
66 (self management or self managing).ti,ab,kf. (8859)
67 (patient$1 adj3 manag$).ti,ab,kf. (103052)
68 health education/ (52650)
69 education.ti,ab,kf. (312282)
70 self care/ or self administration/ or self medication/ (37387)
71 self care.ti,ab,kf. (10701)
72 self monitor$.ti,ab,kf. (4794)
73 self treat$.ti,ab,kf. (1208)
74 (behavio?r$ adj3 (chang$ or modif$ or condition$)).ti,ab,kf. (53656)
75 Patient Satisfaction/ (59876)
76 (patient$ adj3 (experience$ or attitude$ or view$1 or satisfaction$)).ti,ab,kf. (115559)
77 Qualitative research/ (20773)
78 exp Questionnaires/ (316878)
79 exp Interviews as Topic/ (57015)
80 qualitative.ti,ab,kf. (126369)
81 (interview$ or questionnaire$ or focus group$).ti,ab,kf. (513871)
82 or/65-81 (1292227)
83 4 and 64 and 82 (2394)
84 randomized controlled trial.pt. (385321)
85 controlled clinical trial.pt. (89633)
86 randomi?ed.ab. (365820)
87 placebo.ab. (158443)
88 clinical trials as topic.sh. (171898)
89 randomly.ab. (219859)
90 trial.ti,ab. (375660)

91 or/84-90 (1057879)
92 83 and 91 (570)
93 exp animals/ not humans/ (3992529)
94 ((editorial or news or case reports) not randomized controlled trial).pt. (2224741)
95 case report.ti. (161526)
96 92 not (93 or 94 or 95) (569)
97 limit 96 to english language (547)
98 remove duplicates from 97 (504)

Key to Ovid symbols and commands:

$	truncation symbol
?	wildcard symbol
ti,ab,kf,	searches are restricted to the title, abstract, keyword heading word fields
/	searches are restricted to the subject heading field
sh	searches are restricted to the subject heading word field
exp	the subject heading is exploded
*	the subject heading is searched as a major descriptor only
pt.	search is restricted to the publication type field
or/1-3	combine sets 1 to 3 using OR
